# Correction: Sulfate activation of wheat straw ash to enhance the properties of high-performance concrete with recycled aggregates and waste tire steel fibers

**DOI:** 10.1371/journal.pone.0316659

**Published:** 2024-12-27

**Authors:** Fadi Althoey, Osama Zaid, Khaled Mohamed Elhadi

There are errors in the Funding statement. The correct Funding statement is as follows: The authors extend their appreciation to the Deanship of Research and Graduate Studies at King Khalid University for funding this work through the Large Research Project under grant number RGP2/213/45.
